# Future-Oriented Biomaterials Based on Natural Polymer Resources: Characteristics, Application Innovations, and Development Trends

**DOI:** 10.3390/ijms26125518

**Published:** 2025-06-09

**Authors:** Oscar Amponsah, Prince Sungdewie Adama Nopuo, Felista Adrehem Manga, Nicole Bianca Catli, Karolina Labus

**Affiliations:** 1 Department of Micro, Nano and Bioprocess Engineering, Faculty of Chemistry, Wrocław University of Science and Technology, ul. C.K. Norwida 4/6, 50-373 Wrocław, Poland; 282347@student.pwr.edu.pl; 2 Department of Chemical Engineering, Faculty of Chemical Sciences and Technology, University of Castilla-La-Mancha, C. Altagracia, 50, 13005 Ciudad Real, Spain; princesungdewieadama.nopuo1@alu.uclm.es; 3 Department of Chemical Process Engineering and Technology, Faculty of Chemistry, Wrocław University of Science and Technology, ul. C.K. Norwida 4/6, 50-373 Wrocław, Poland; 282484@student.pwr.edu.pl; 4 Department of Separation Science, School of Engineering Science, Lappeenranta-Lahti University of Technology, Mukkulankatu 19, 15210 Lahti, Finland; nicole.catli@student.lut.fi

**Keywords:** natural-based materials, functional properties, innovative applications, future directions

## Abstract

This review comprehensively explores natural polymer-based materials, focusing on their characteristics, applications, and innovations across different sectors, including medicine, the environment, energy, textiles, and construction. With increasing concern about resource depletion and pollution, biomaterials offer a sustainable alternative to fossil-derived products. The review highlights polysaccharide-based and protein-based biomaterials, as well as others, such as polyisoprene, rosin, and hyaluronic acid. Emphasis is laid on their compositions and attractive characteristics, including biocompatibility, biodegradability, and functional versatility. Moreover, the review deeply discusses the ability of natural polymers to form hydrogels, aerogels, films, nanocomposites, etc., enhanced by additives for innovative applications. Future development trends of biomaterials in biomedicine, sustainable materials, environmental biotechnology, and advanced manufacturing are also explored. Their growing potential in these sectors is driven by research advances in emerging technologies such as 3D bioprinting, nanotechnology, and hybrid material innovation, which are proven to enhance the performance, functionality, and scalability of biopolymers. The review suggests several strategies, including improvement in processing techniques and material engineering to overcome limitations associated with biomaterials, thereby reinforcing their suitability and role in a circular and sustainable economy.

## 1. Introduction

Resource depletion, climate change, and waste accumulation are environmental issues that are largely becoming more relevant nowadays. Material utilization has tripled within the last 50 years with a steady growth of about 2.3% per year, and material source extraction could reach 160 billion tonnes at the current rate. This is especially concerning as resource extraction and processing are responsible for 55% of global greenhouse gas emissions [1]. These issues, along with consumer demand for eco-friendly solutions, are the key drivers for the intensified global push for the industry’s reduced reliance on and preference for petroleum-based materials. Bio-based materials, defined to be materials that are fully or partially of biological origins [2], are central to the shift from a linear economy to a circular bioeconomy which prioritizes the utilization of renewable biological resources for the development of sustainable products and services [3]. The suitability of bio-based materials as a sustainable alternative to fossil fuel-based materials is further underscored by the natural source abundance, renewability, and low-impact production processes, contributing to an overall lower carbon footprint [4]. Furthermore, many bio-based materials are biodegradable, which can significantly reduce the environmental impact linked with plastic waste [5,6].

Their application spans a multitude of industries including medical, packaging, energy storage, textiles, and construction [7]. The urgent need for bio-based materials drives technological innovations, leading to the significant advancements witnessed in the last decade by the material sector. For instance, a common polysaccharide like starch has been transformed into bio-based materials like bioplastics and hydrogels which can be extensively used in the biomedical sector as a drug delivery system, implants, and tissue engineering [8,9], in the food sector as a biodegradable and recyclable food packaging alternative to single-use plastics [10], as well as for bioremediation [11]. Natural fibers such as hemp or flax blended with biodegradable resins form biocomposites that find their usability in industries requiring materials with enhanced mechanical, thermal, and functional characteristics such as in automotives and sports [12,13]. The valorization of agricultural waste, such as pomelo peel, into high-performance carbon materials for energy storage applications further illustrates the versatility of biological resources in creating sustainable solutions [14]. The construction industry also benefits from the bio-based sector through innovations like flame-retardant additives derived from sugarcane bagasse for insulation materials [15] and concrete from hemp [16]. These innovations reflect the growing acknowledgment of bio-based materials as sustainable and practical alternatives across diverse industries, signaling a fundamental shift toward greener production practices.

Despite their vast potential, the widespread adoption of bio-based materials is not without challenges, particularly in the aspect of land use and associated environmental impacts. A comprehensive assessment of a bio-based material’s environmental footprint is essential to achieve an accurate and unbiased comparison with its fossil fuel-based counterpart, typically accomplished with the aid of life cycle assessments (LCA). Weiss et al. [17] emphasized the importance of such evaluations, noting that drawing general conclusions about bio-based materials is difficult [17]. While significant reductions in energy consumption and greenhouse gas (GHG) emissions are observed, bio-based materials, unfortunately, can contribute to increased eutrophication and stratospheric ozone depletion, primarily due to the use of nutrients and chemicals during biomass cultivation. Additionally, land use impacts such as biodiversity loss, soil nutrient depletion, land conversion, and erosion are critical considerations. Bio-based materials can likewise face economic and logistical barriers. Feedstock seasonality, avoiding negative impacts on food security, and supply chain limitations can severely limit scaled-up production. Moreover, the specialized processing techniques and raw materials further inflate the cost and complexity of manufacturing these bio-based materials [18].

However, these constraints should not be viewed as reasons to abandon the shift to bio-based materials in favor of conventional petroleum-based products but rather as opportunities for innovation and progress. This is evident and will be presented in detail in this review, which comprehensively explores the current state of bio-based materials, examining their key characteristics, sources, and diverse applications. It also highlights innovations across multiple sectors, from packaging and automotive to textiles and construction, providing insights into how bio-based materials are transforming industries. In analyzing the latest developments and trends, this article emphasizes the role of bio-based materials in advancing a more sustainable and circular bioeconomy. The field of biomaterials is rich with potential and offers numerous research opportunities in both material science and production technologies. With the right policies and investments, the bio-based materials sector can drive significant academic and economic growth while supporting global sustainability goals. In that regard, ultimately, this review aims to serve as a valuable resource for researchers, industry professionals, and policymakers advocating for the development and widespread adoption of bio-based materials as a central pillar for sustainable material solutions.

## 2. Characteristics of Biomaterials

**Figure 1:**
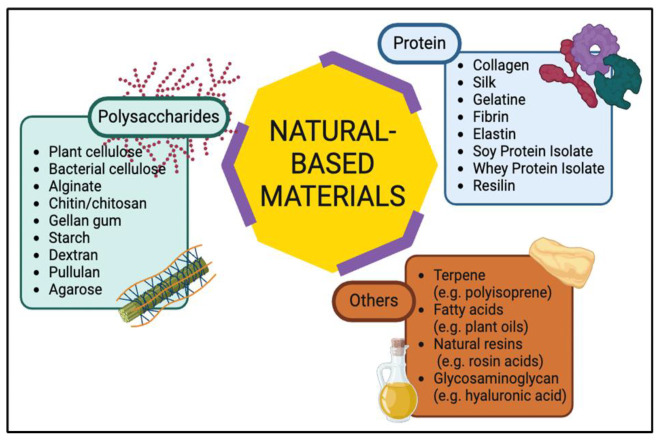
Classification of natural-based materials [7,19,20,21,22] (created in https://BioRender.com).

Natural-based polymeric materials, classified mainly as proteins or polysaccharides, have gained great attention in recent years, mainly due to their potential to replace petroleum hydrocarbon-derived plastics in a variety of applications. These biomaterials have exhibited good chemical stability, structural versatility, biocompatibility, and high availability with diverse applications in areas such as tissue engineering, drug delivery and wound healing, energy materials fabrication, and processing. Such materials are usually obtained from plants, animals, fungi, bacteria, and algae sources and can also be engineered to improve their structural and functional properties. Some common examples of biomaterials are classified as shown in Figure 1.

### 2.1. Polysaccharide-Based Materials

#### 2.1.1. Cellulose

**Figure 2:**
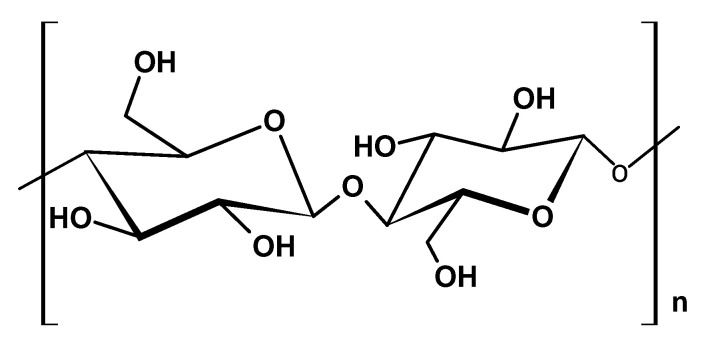
Chemical structure of cellulose.

Cellulose is considered the most abundant natural biopolymer, with an annual production of 250 billion tonnes [23]. It features an overall crystalline structure composed of β-D-glucose units linked with β-1,4-glycosidic bonds and is rich in reactive hydroxyl groups (Figure 2). Cellulose can be derived from either plant sources (plant cellulose (PC)) or bacterial sources (bacterial cellulose (BC)). PC, the primary structural component of plant cell walls, is obtained from lignocellulosic sources. On the other hand, Tanskul et al. [24] reported that BC can be isolated from over 59 strains of bacteria from ripe fruits and vegetables, including Gram-negative bacteria (*Gluconacetobacter xylinus*, *Agrobacterium*, and *Rhizobium*) and Gram-positive bacteria (*Sarcina)* [24].

PC and BC differ significantly in terms of fiber properties and purity. PC fibers have diameters of 13–22 µm, typically contain impurities like residual hemicellulose, and are less crystalline, with a crystallinity index of around 44–65% due to the high proportion of cellulose I β [25,26]. In contrast, BC is highly pure and free of biogenic contaminants [27], which grants it superior physicochemical properties. These include high specific surface area (fibers with diameter 20–100 nm and high aspect ratio), porosity, mechanical strength (Young’s modulus 15–18 GPa), degree of crystallinity (up to 80%) and polymerization (1000–20,000), high water-holding capacity (over 100 times of its weight), and biodegradability [19,28].

Cellulose is versatile and has applications spanning multiple industries such as pulp and paper, food, textiles, biocomposites and bioplastics, biomedical, pharmaceuticals, cosmetics, and nanotechnology [26,29]. Particularly, Ventura-Cruz and Tecante [30] highlighted the potential of agro-industrial residues as sustainable sources of plant cellulose nanofibers and nanocrystals, which can be used for a multitude of nanotechnological applications. BC-based hydrogels have a nanofiber structure, allowing them to absorb, store, and desorb large amounts of water [30]. Some functional BC-based composites include BC/chitosan composite for wound dressing materials and BC/alginate for tissue engineering [31,32]. BC’s dietary fiber properties are utilized to produce nata-de-coco, a juicy and chewy dessert from the Philippines [18].

#### 2.1.2. Alginate

**Figure 3:**
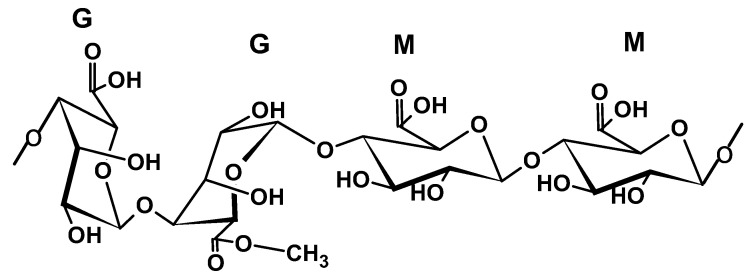
Chemical structure of alginate.

Alginate is an anionic polysaccharide known for its hydrophilic properties. It can be extracted from marine plants (comprising up to 40% of dry weight) and, to a lesser extent, from bacteria. Large-scale production comes from brown algae species, including *Laminaria hyperborea*, *Ascophyllum nodosum*, and *Macrocystis pyrifera*, while small-scale production involves bacterial species such as *Azotobacter vinelandii* and *Pseudomonas aeruginosa* [33]. Structurally, it contains linear blocks of (1,4)-linked β-D-mannuronic acid (M) and α-L-guluronic acid (G) monomers (Figure 3) arranged in different block segments: homopolymeric blocks of G or M residues or heteropolymeric blocks of alternating MG residues. Its high reactivity is due to its numerous reactive sites, namely, two unstable bonds (1,4 O-glycosidic and internal glycolic bonds) and carboxylic acid and hydroxyl functional groups [34].

Alginate exhibits key biological properties such as non-toxicity, biocompatibility, and biodegradability that make it an ideal material for producing hydrogels, injectable gels, microspheres, porous scaffolds, films, and fibers. These materials have diverse applications in various fields, like regeneration medicine (for the repair and regeneration of various tissues and organs such as skin, cartilage, and bone), nutrition supplements, semi-permeable separation, and biodegradable packaging [35].

#### 2.1.3. Chitin

**Figure 4:**
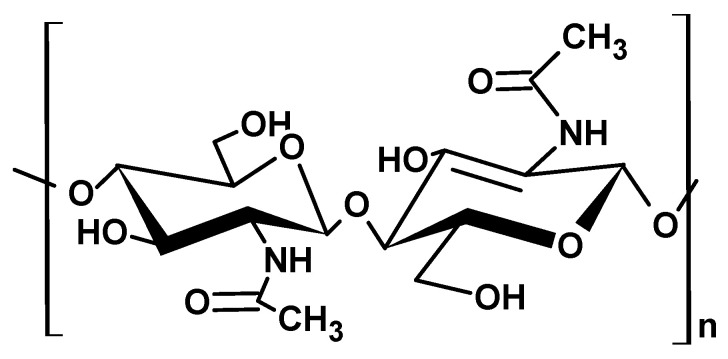
Chemical structure of chitin.

Chitin is a polysaccharide generally obtained from the exoskeleton of crustaceans and other chitin-containing sources like cephalopods, protozoans, coelenterates, and seaweed. [36]. As reported by Gadgey et al. [37], chitin biopolymer is a high molecular weight (120 kDa), amorphous solid that has a density ranging from 0.18 to 0.33 g/cm^3^, high to low viscosity, and solubility in dilute acidic solvents but remains insoluble in water, alkali, and organic solvents. Chemically (Figure 4), chitin biopolymers have linear polyamine and reactive amino groups that exhibit affinity toward negatively charged surfaces. Its degree of acetylation ranges from 70–90% and has a high charge density at pH < 6.5 [37]. Chitin exhibits three polymorphs, namely α, β, and γ, based on the chain orientations. The α-chitin has been reported to be most abundant in shellfish shells and the most stable among the three. However, β- and γ- chitin possess the ability to transform into α-chitin under favorable conditions [38].

Studies have found chitin biopolymers to possess excellent biological properties, including non-toxicity, biocompatibility, and antioxidant, anticarcinogen, and antitumor properties upon application [38,39]. However, its highly insoluble nature translates to poor biodegradability properties hence requiring further processing or the use of functional additives [38].

#### 2.1.4. Chitosan

**Figure 5:**
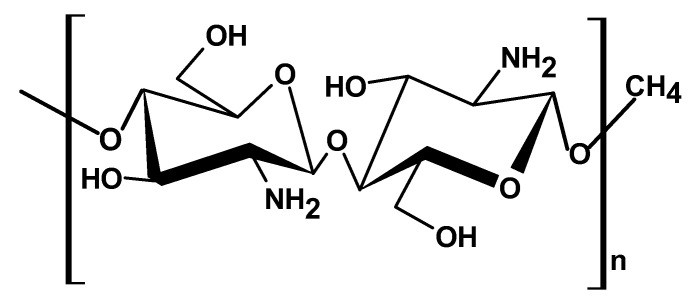
Chemical structure of chitosan.

Chitosan, a chitin-derived polysaccharide, is composed of linear β-(1-4)-2-amino-2-deoxy-d-glucopyranose repeating units. It is considered a copolymer of N-acetylglucosamine and N-glucosamine units randomly or block-distributed throughout the biopolymer chain (Figure 5) [6,40]. The polymer chain contains amino functional groups in the deacetylated units at C-2 and hydroxyl groups at C-3 and C-6 positions, which confer chitosan with excellent reactivity and versatility [5,6].

The physiochemical and biological properties of chitosan depend on the initial source and composition of the chitin, as well as the processing method [41], but in general, it possesses excellent biological properties such as biodegradability, biocompatibility, and antimicrobial, antioxidant, and anti-inflammatory activity [40,41]. Chitosan is a crucial material across various sectors, especially in the biomedical field. Its cationic character, arising from the presence of amino groups, affords it with key properties essential for drug delivery applications such as controlled drug release, mucoadhesion, in situ gelation, transfection, permeation enhancement, and efflux pump inhibitory [37].

#### 2.1.5. Gellan Gum

**Figure 6:**
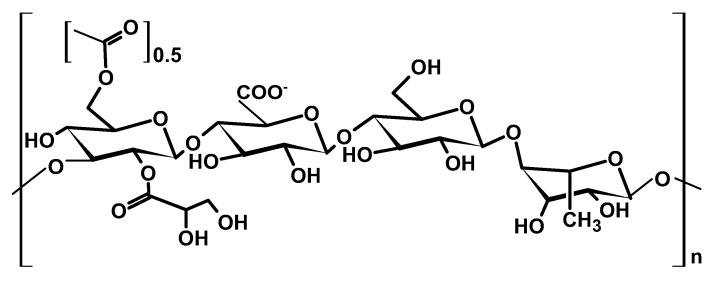
Chemical structure of gellan gum.

Gellan gum (GG) is a tetrasaccharide natural biopolymer with a linear structure of repeating units of two subunits of β-D-glucose and one subunit each of β-D-glucuronate and α-L-rhamnose (Figure 6) [42].

The bacterium *Sphingomonas paucimobilis* secretes GG, which is then extracted from the post-culture fluid for commercial purposes. Its versatility allows for tailoring physicochemical properties to suit a wide range of industrial applications [43]. It has exceptional biocompatibility and biodegradability characteristics, making it a common additive in biomedical products for enhanced compatibility and minimizing adverse reactions [44]. Furthermore, the presence of hydroxyl groups in the polymer chains allows it to form salt complexes, rendering it a unique material for electrolytes in batteries, solar cells, and other electrochemical applications. GG’s ability to form a three-dimensional crosslinked hydrogel under optimized process conditions makes it ideal for a wide range of biomedical and pharmaceutical applications, as hydrogels facilitate the controlled release of therapeutic compounds in drug delivery [45,46,47].

#### 2.1.6. Starch

**Figure 7:**
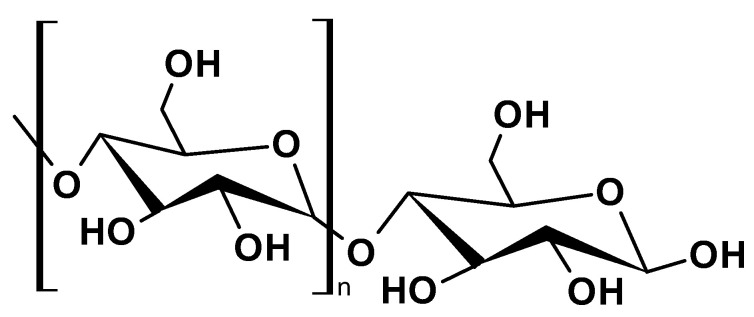
Chemical structure of starch.

Starch is the most abundant storage polysaccharide in plants, and is primarily sourced from corn, wheat, potatoes, and rice. Chemically, starch is a homopolymer composed of repeated glucose units (Figure 7). It exists in two forms: amylose and amylopectin [48]. Amylose is a linear polysaccharide composed of 500–2000 glucose units linked by α-1-4 glycosidic bonds and constitutes 20–30% of starch. Amylopectin is a branched polysaccharide with chain lengths of 1,000,000 glucose units linked by α-1-6 glycosidic bonds, making up 70–80% of starch [49].

Starch exhibits unusual physicochemical properties that limit its industrial applications, such as insolubility in cold water and viscosity fluctuation during thermal processing. However, its other properties, such as low cost, low density, large surface area, and porosity, make starch attractive for novel bioproduct development. The biological aspects, including renewability, biocompatibility, degradability, and non-toxicity, make it invaluable for biological applications in the medical and pharmaceutical fields [50]. Nanocomposites (starch/hydroxyapatite), hydrogels, foams, films, and aerogels can be synthesized from starch, with applications in ophthalmic drug delivery, infectious disease treatment, regenerative medicine, and tissue engineering [48].

#### 2.1.7. Dextran

**Figure 8:**
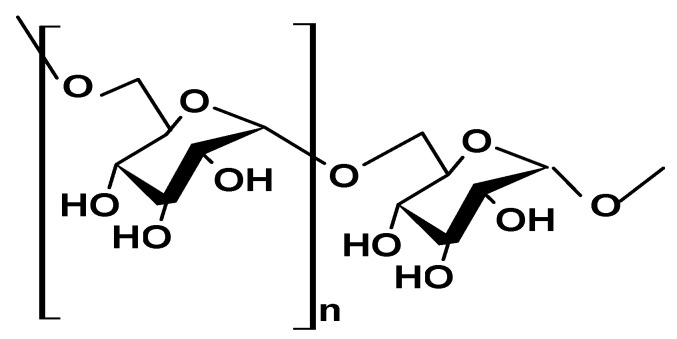
Chemical structure of dextran.

Dextran, a natural exopolysaccharide secreted by lactic acid bacteria, has a long chain of D-glucose molecules connected by α-(1→6) bonds, but may also have branching points where D-glucose molecules are linked by α-(1→4), α-(1→3), or α-(1→2) bonds (Figure 8). Its molecular weight can vary widely, ranging from around 1 up to 2300 kDa [51,52,53].

Dextran has unique solubility, viscosity, and thermal and rheological characteristics that make it a valuable resource in various industries, including food, pharmaceuticals, and biotechnology research. For example, Kavlak et al. [54] prepared a dextran–polymethacrylamide (PMAM) blend using a solution casting method and explored the dynamic mechanical properties and thermal transitions of biocompatible dextran (T10 and T40) as a viable material for biomedical applications [54].

#### 2.1.8. Pullulan

**Figure 9:**
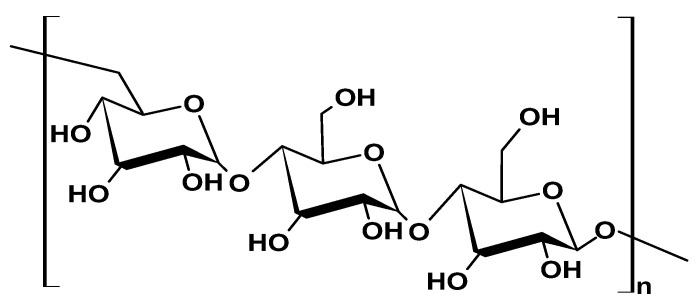
Chemical structure of pullulan.

Pullulan is a highly soluble, tasteless, odorless, edible, and biodegradable fungal exopolysaccharide produced by *Aureobasidium* spp. under aerobic conditions and a carbon-rich substrate [55,56,57]. Its numerous advantages, including heat resistance, non-carcinogenicity, non-toxicity, non-immunogenicity, and wide viscosity range give it significant relevance in the industrial setting. Its structure constitutes maltotriose units connected by α-1,4 and α-1,6 glycosidic bonds in an unbranched sequence (Figure 9) [58].

Pullulan has a relatively lower viscosity compared to other polysaccharides, with a molecular weight of 40–600 kDa and a melting point range of 250–300 °C [57,59]. It is used to fabricate a wide range of biomaterials for applications, including cholesterol-bearing nanogels for wound healing [60,61], hydrogels for vascular cell engineering [62], gels for skin tissue regeneration [61], hard capsules for in vitro drug delivery [63], or pullulan–dextran microbeads for bone repair [55].

#### 2.1.9. Agarose

**Figure 10:**
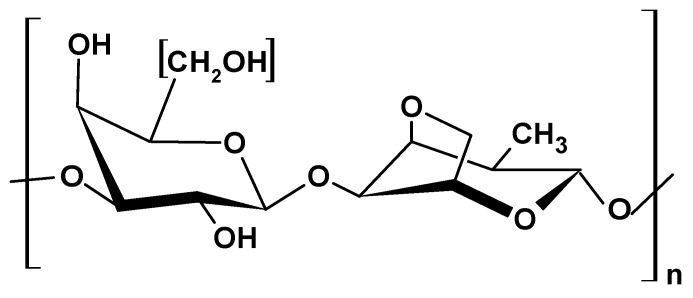
Chemical structure of agarose.

Agarose is a natural polysaccharide obtained from marine sources, usually red algae, comprised of alternating units of β-D-galactopyranosyl and 3,6-anhydro-α-L-galactopyranosyl [64]. It is considered a thermally gelling alternating copolymer of β-1,3-linked D-galactose and α-1,4-linked 3,6-anhydro-α-L-galactose fragments (Figure 10).

Agarose has a unique temperature-sensitive property, allowing its transition from a water-insoluble gel-like state to a water-soluble state. This makes it an ideal material for cell encapsulation, as proven by Nilsson [65], who successfully immobilized and entrapped suspension-grown cells within polymer microbeads, paving the way for new possibilities in cell culture and research with agarose [65]. Consequently, advanced research has been conducted, among which Karoubi et al. [66] made a significant breakthrough by developing a single-cell agarose hydrogel microcapsule. Their findings revealed that agarose microcapsules can efficiently support the survival of transplanted cells, achieved by striking a balance between mass transfer and metabolic requirements, demonstrating the great potential of agarose as a cell-delivery system [66].

### 2.2. Protein-Based Materials

#### 2.2.1. Collagen

**Figure 11:**
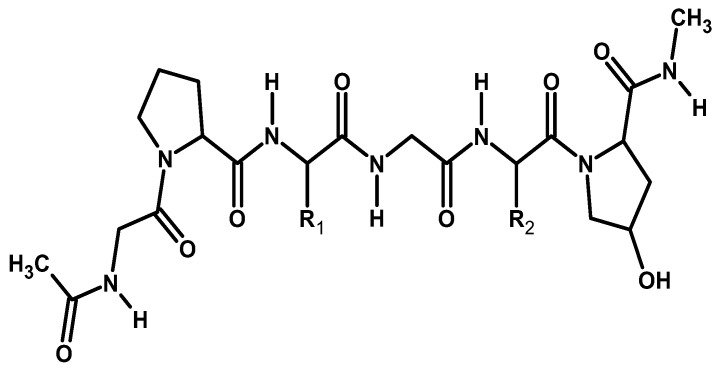
Chemical structure of collagen.

Collagen is a widely distributed class of protein that originates from a wide range of animals, including mammals and chordate classes. It can be sourced from animal skin (pigs, cattle, sheep), waste leather material, Achilles tendons, rat-tail tendons, fish skin, or through a recombinant protein production system. This fibrous protein is the major constituent of skin and bone, accounting for roughly 25% of the total dry weight in mammals [67]. Collagen’s structure is composed of three protein chains (alpha (α) chains) that form a distinctive triple helix structure. Each α chain contains approximately 1000 amino acids and has a molecular weight of about 100 kDa, based on the repeating sequence of specific amino acids Gly-Xaa-Yaa (Figure 11). Gly is the smallest amino acid, with a hydrogen atom as its side chain, allowing it to fit at the center of the triple helix without causing steric hindrance while the Xaa and Yaa positions are predominantly filled by proline and 4-hydroxyproline [68].

Collagen’s stability, plasticity, and viscosity are temperature-dependent, with a degradation temperature ranging from 5 to 50 °C. Its ability to bind ligands such as integrins and low-density lipoproteins helps in cell signal transmission. Moreover, it possesses biological properties that include low toxicity, low antigenicity, easy absorption by the body, biocompatibility, and biodegradability, which contribute to its wide applications in medicine, pharmacology, and implantology [68,69]. Collagen has found several applications in tissue engineering including Type I collagen widely used in bone repair, Type II in cartilage regeneration, and Type I, III, and VI in skin tissue regeneration as a naturally occurring matrix or composites with improved strength. Its biological activity is maintained upon application in forms such as hydrogel, freeze-dried sponges, collagen membranes or films, creams, injectable preparations, coatings, and scaffolds [70].

#### 2.2.2. Gelatin

**Figure 12:**
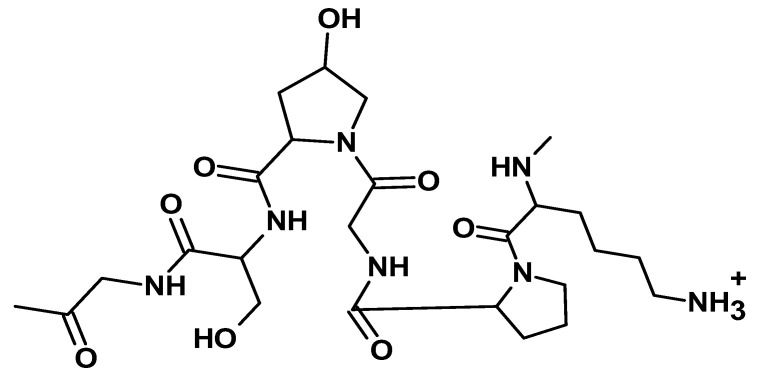
Chemical structure of gelatin.

Gelatin is a biopolymer of animal origin, primarily obtained from animal bones, cartilage, ligaments, and skin. It is often referred to as hydrolyzed collagen, as it is obtained through the incomplete hydrolysis of collagen [71]. During the hydrolysis process, collagen undergoes structural breakdown to yield a mixture of peptides of varying molecular weights (Figure 12) [72].

The physicochemical properties of gelatin are largely dependent on its sources; gelatin from mammals exhibits superior properties compared to fish gelatin, which in turn outperforms insect-derived gelatin [72]. In general, gelatin is soluble in warm water, forms a gel upon cooling, and melts at normal body temperature, with its viscosity dependent on concentration and temperature. It is biocompatible, biodegradable, and non-toxic, which determines its wide range of practical applications [73]. Among others, gelatin is one of the widely used ingredients in food and non-food industries for improving thickening, stabilization, gelation, and emulsification purposes [71]. Gelatin-based hydrogels have wide applications in the biomedical sector, including drug delivery, tissue engineering, tissue adhesives, wound dressings, and wearable devices [74]. These can likewise be utilized in environmental applications such as contaminant removal during wastewater treatment [75,76].

#### 2.2.3. Silk

**Figure 13:**
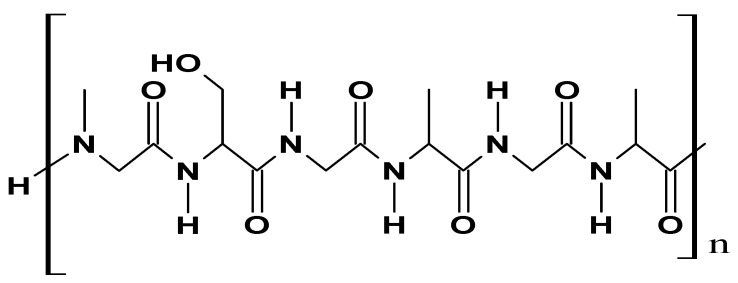
Chemical structure of silk.

Silk is classified as a naturally occurring protein-based polymer usually extracted from spiders and the silkworm *Bombyx mori*. Silk has gained significant interest because of its ease of handling, biocompatibility, and environmentally friendly character [77]. Structurally (Figure 13), silk polymer is made of two main proteins: silk fibroin—the core structural protein—and sericin, a glue-like globular protein coating the fibroin [77,78].

Silkworm silk is semi-crystalline (62–65% crystallinity) and demonstrates thermal stability at temperatures below 100 °C. Its excellent mechanical properties, biocompatibility, biodegradability, and good versatility in terms of structural adjustment make it a suitable biomaterial [79]. For instance, silk is used in tissue engineering and as a matrix for wound healing as well as in the fight against cystitis, edema, impotence, epididymitis, and cancer [78]. Moreover, their anti-inflammatory and anti-tumfacient properties make them suitable for treating acute sinusitis and tonsillectomy. Silk-based hydrogels have also been intensively examined as drug-delivery systems [77].

#### 2.2.4. Fibrin

**Figure 14:**
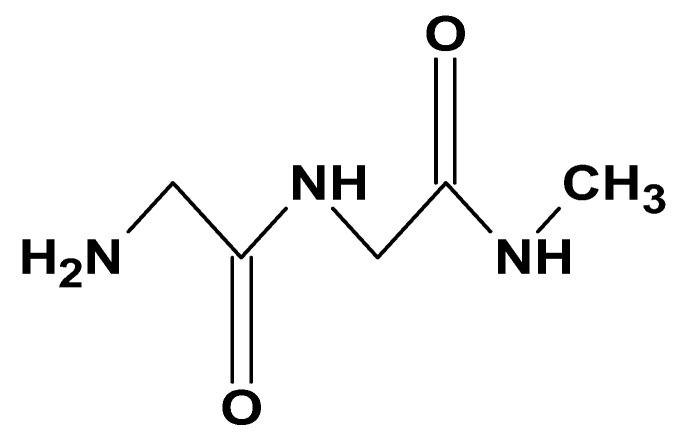
Chemical structure of fibrin.

Fibrin is a protein-based biopolymer obtained from fibrinogen through a cascade of enzymatic reactions activated by thrombin [80]. Fibrinogen, a major component of the blood, is a long glycoprotein with a molecular weight of about 340 kDa. It is composed of a dimer formed by three disulfide-linked polypeptide chains, namely Aα, Bβ, and γ, having molecular weights of 66.5 kDa, 52 kDa, and 46.5 kDa, respectively [81]. Fibrinogen’s structure comprises two globular D regions and one central E region, each containing portions of α-helical coiled coils (Figure 14).

Fibrin polymers have excellent mechanical strength, good elasticity, and mesh-like structures and are considered biodegradable and biocompatible [82,83]. These characteristics make them ideal for a range of applications, particularly in the biomedical industry. Some products from fibrin include glues, scaffolds, hydrogels, gels, microbeads, composites, and sealants, each with specific usage in the biomedical field [84].

#### 2.2.5. Elastin

**Figure 15:**
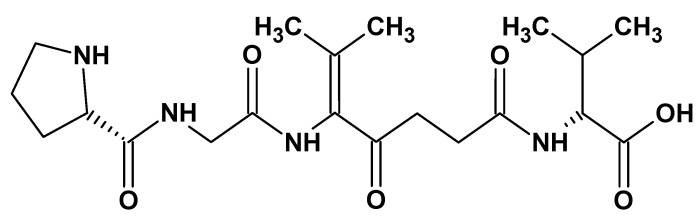
Chemical structure of elastin.

Elastin is an elastomeric, insoluble, and fibrous protein that forms the internal core of elastic fiber, constituting the extracellular matrix, and is mainly present in organs and tissues such as skin, ligaments, bladder, blood vessels, cartilage, and lungs [85,86]. It is synthesized during elastogenesis from tropoelastin [85], a protein precursor secreted by elastogenic cells (e.g., endothelial cells, smooth muscle cells, and fibroblasts), and has a molecular weight of about 60 kDa [87]. Structurally (Figure 15), elastin in the form of elastic fibers is composed of two parts: an inner core of amorphous crosslinked elastin, which constitutes about 90%, and an outer microfibrillar mantle [86].

Elastin biopolymers are hydrophobic and insoluble and thereby can resist acid and alkali degradation. It exhibits rubbery properties in the presence of water, with a low elastic modulus [87]. Elastin is considered a biodegradable, biocompatible, and low immunogenicity biopolymer with excellent elasticity, resilience, durability, and longevity [88,89]. Due to its excellent physicochemical and biological properties, elastin has found applications in various sectors. Among others, it is used in the fabrication of elastin-like polypeptides used as scaffolds for tissue engineering [90] and hydrogel-based drug delivery systems [86].

#### 2.2.6. Soy Protein

Soy protein is obtained from soybeans processed in the edible oil industry and is regarded as a by-product of the edible oil extraction process [91,92]. There are three main types of soy protein products, categorized by their varying concentrations (ranging from 50% to 90%) and commercially available as soy flour, soy protein concentrate, and soy protein isolate (SPI), each with distinct differences in protein content [93]. Soy protein is a blend of albumins and globulins, with the majority (90%) being storage proteins, mainly consisting of 7S (β-conglycinin) and 11S (glycinin) globulins [94]. The molecular weights and isoelectronic point values of the 11S and 7S differ significantly, with values of 360 kDa and 6.4 and 175 kDa and 4.8, respectively [95]. Soy protein is a relatively stable globular protein and contains a variety of functional groups such as -NH_2_, -OH, -SH, and -COOH, which facilitate convenient modification (chemical or physical) and combination with other biopolymers to produce materials of higher value. Soy protein has many useful applications in sectors including food packaging or edible films as it complies with food-grade standards and has a long storage life [93,96]. It is likewise commonly used in the production of hydrogels, adhesives, plastics, films, coatings, and emulsifiers, and is considered a material with high potential for biotechnological and biomedical applications [97].

#### 2.2.7. Whey Protein

Whey accounts for 20% of the total protein in milk and is essentially the translucent, fluid byproduct of the cheese-making process [98]. Whey proteins are further separated from whey and purified through various techniques, resulting in different concentrations of whey proteins. It comes in three primary products, varying in protein content: whey powder (11–14.5% protein), whey concentrates (25–89% protein), and whey isolate (>90% protein) [98]. WP is composed primarily of β-Lactoglobulin (β-La) (50–60%), a small globular protein with 162 amino acids and a molecular weight of approximately 18.3 kDa [99]. It also contains α-Lactalbumin (α-La; 14.2 kDa) at 20–25% [100], 1.2–1.5% of bovine serum albumin (BSA; 66.5 kDa), and <10% of lactoferrin (78–80 kDa) [101,102]. A strong hydrogen interaction between calcium ions and α-La results in a stable molecular structure, enhanced binding capacity, heat stability, and non-gelling characteristics.

Whey and whey protein hold significant potential, with Smithers [103] using the phrase “gutter to gold” to describe the transformation of whey into its derivatives. Extensive research conducted to fully ascertain the enormous advantages of whey protein revealed its versatility and applicability in a variety of fields, including pharmaceuticals, manufacturing, food and beverages, and therapeutic applications [104]. Researchers are currently exploring the sustainable valorization of whey, including conversion into bioethanol and other value-added products through fermentation. For instance, recent reviews highlight the advances in cheese whey biotransformation into bioplastics and natural colors and flavors as well as bioethanol production using engineered yeast and co-cultures, obtaining high bioethanol yields at optimal conditions [105].

#### 2.2.8. Resilin

Resilin, obtained from a wide range of arthropods, is an endogenous extracellular matrix protein composed primarily of amino acids with a high proportion of acidic residues and a low proportion of nonpolar residues. This composition results in a low isoelectric point and a strong hydrophilic nature [106]. It is the most efficient known elastic protein as it works practically as a perfect isotropic rubber, exhibiting long-range reversible elasticity and exceptional resilience [106,107]. Hydration transforms resilin into a hydrogel (at 50–60% water content and neutral pH) with significantly altered physical properties. This transformation enables resilin to switch between two distinct states: a flexible, rubber-like material when hydrated but otherwise a rigid, glassy polymer [108]. Much research has been conducted on resilin for various applications, including effective energy storage and biomedical purposes, especially tissue engineering, due to its reversible elasticity, high-frequency responsiveness, hydrophilic capacity, self-assembly behavior, and fluorescent properties. Lihui and Wen [109] found that human mesenchymal stem cells formed more extensive stress fibers on resilin-based hydrogels with RGD sequences, enhancing cell attachment and expansion [109]. On the other hand, resilin-based material (RZ10) developed by Renner et al. [110] displayed an unconstrained compressive modulus comparable to that of human cartilage, making it suitable for tissue engineering applications [110]. Truong et al. [111] also created a pH-responsive biosensor surface by attaching a resilin-mimetic protein to gold nanoparticles in a compact state. This innovative design leveraged the protein’s fluorescence capabilities and nanoparticle stabilizing properties, enabling precise control over nanoparticle growth rates [111].

### 2.3. Other Natural-Based Materials

#### 2.3.1. Polyisoprene

**Figure 16:**
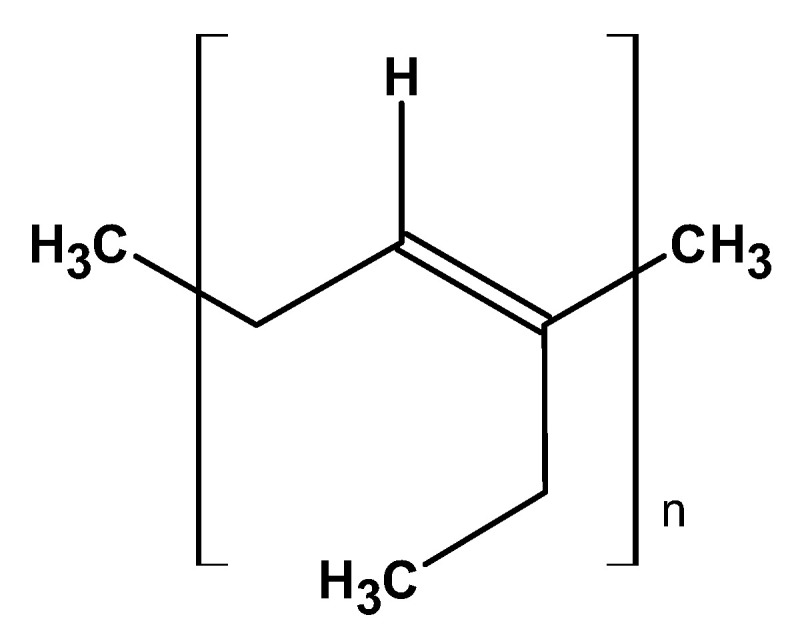
Chemical structure of polyisoprene.

Polyisoprene is a polymer consisting of chains of isoprene monomers, primarily of cis-1,4-polyisoprene (Figure 16).

It is a natural rubber obtained from the sap of *Hevea brasiliensis* (rubber tree). This material is known for its excellent elasticity and resilience, making it an attractive material for different applications, particularly in tire manufacturing, footwear, and various latex products [112].

#### 2.3.2. Plant Oils

Plant oils are increasingly gaining attention in the field of sustainable biomaterials due to their versatility in application, particularly those derived from vegetable sources like soybean, olive, palm, and sunflower. They can be valorized into several polymeric biomaterials with biodegradable and biocompatible properties well-suited for applications in the biomedical sector such as drug delivery, tissue engineering, and scaffolding for cell growth. Valorization includes functionalizing these oils to enhance their properties through acylation, transesterification, metathesis, and epoxidation reactions. These processes transform the triglycerides into polymerizable monomers, allowing for the formulation of materials with specific mechanical and chemical characteristics tailored for biomedical applications [113].

#### 2.3.3. Rosin

**Figure 17:**
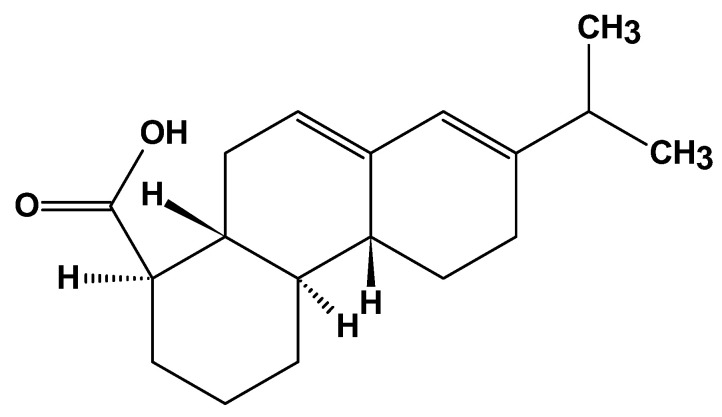
Chemical structure of rosin.

Rosin, a solid form of resin, is an amber-colored, transparent, glass-shaped natural biodegradable polymer obtained from pine trees and other plants. It primarily consists of resin acids, especially abietic acid (Figure 17) [114].

It has a variety of applications, including as a glazing agent in medicines and chewing gum, as well as in paper-making chemicals, paints, adhesives, and as a flux in soldering. Rosin acids readily crystallize, leading to issues like increased viscosity and reduced storage life; hence, in practical applications, carboxylic acids and their salts can be used as crystallization inhibitors for rosin-based materials [115].

#### 2.3.4. Hyaluronic Acid

**Figure 18:**
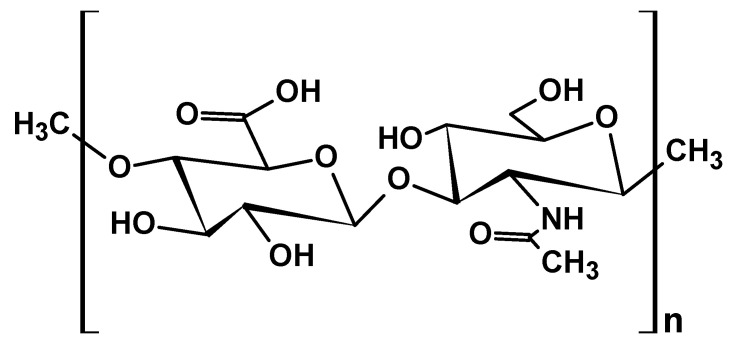
Chemical structure of hyaluronic acid.

Hyaluronic acid (HA) is a natural biopolymer made up of repeating disaccharide units formed by D-glucuronic acid and N-acetylglucosamine molecules connected through β-(1-4) and β-(1-3) glycosidic bonds (Figure 18).

HA, a part of the glycosaminoglycan (GAG) family, is classified as a mucopolysaccharide. Its remarkable ability to retain large quantities of water makes it a key ingredient in aesthetic medicine, boosting tissue hydration and resistance to mechanical stress. It is biocompatible, biodegradable, and has broad availability. As such, it has been widely utilized across various medical fields, such as oncology, orthopedics, ophthalmology, and aesthetic dermatology [116].

### 2.4. Specific Types of the Functional Forms of Natural Polymer-Based Materials

Natural polymers are engineered into diverse biomaterials and biocomposites with unique properties that allow their inherent biocompatibility, biodegradability, renewability, and versatility to be harnessed for various beneficial applications. These biomaterials are tailored into functional forms, including nanofibers, nanotubes, hydrogels, aerogels, nanoparticles, films, membranes, and 3D porous scaffolds to address global challenges across various sectors. For instance, cellulose-based films are increasingly being developed to replace single-use plastics for sustainable packaging applications [117]. This subsection explores some specific natural polymer-based biomaterials, their unique properties, and fabrication methods.

#### 2.4.1. Nanofibers

Nanofibers derived from bio-based materials are classified as natural or are chemically synthesized, with the former encompassing starch, cellulose, alginate, and chitin, while the latter includes polylactic acid [118]. As the name implies, these fibers are smaller in size, typically having a diameter less than 100 nm, and exhibit excellent surface area-to-volume ratio, mechanical strength, and tunable porosity [119]. Several studies have reported the successful synthesis of nanofibers using various techniques, such as fibrillation [120], melt spinning [121], 3D printing [122], electrospinning [123], microfluidic spinning [124], etc. This class of materials has emerged as a sustainable and versatile bio-based material with a wide range of applications in the packaging industry, biomedical sector, and environmental remediation. For instance, nanofibers have been extensively used as reinforcing materials in the synthesis of bio-based films for packaging materials. The incorporation of nanofibers (e.g., cellulose nanofibers) into biopolymers has been shown to enhance their physicochemical properties, broadening their areas of application [125]. Biopolymer–nanofiber scaffolds have also exhibited excellent performance in cell attachment and proliferation. Recent innovations include the development of bio-ink by incorporating nanofibers into natural pigments for applications such as 3D printing [126].

#### 2.4.2. Nanotubes

The search for renewable materials has driven the development of bio-based nanotubes. This category of nanotubes, including carbon nanotubes (CNTs) derived from biomass, peptide nanotubes, organosilicon polymer nanotubes, cellulose-based nanotubes, chitosan nanotubes, and virus-based protein nanotubes, presents a promising alternative to petroleum-based options [127]. Known for their large surface area, elasticity, high tensile strength, excellent electrical conductivity, and chemical stability, coupled with their biocompatibility and biodegradability, bio-based nanotubes are used in various applications, including biomedicine, sustainable coating, water treatment, and biosensor development. In biomedicine, for instance, Li et al. [128] synthesized a novel siRNA-induced peptide co-assembly nanocarrier as a peptide-based nanocarrier for cancer therapy which exhibited excellent gene silencing efficiency and high biocompatibility both in vitro and in vivo [128]. In sustainable coating, CNTs were incorporated into an epoxidized soybean oil (ESO)-based vitrimer to synthesize a superhydrophobic coating. The coating exhibits excellent self-healing and recyclability properties, giving it excellent anti/deicing and photothermal deicing performance [129]. This highlighted the potential solution for anti-icing and de-icing applications.

#### 2.4.3. Hydrogels

Natural hydrogels are three-dimensional, crosslinked polymeric materials synthesized from biopolymers such as chitosan, starch, alginate, lignin, and gelatin [130]. The hydrogels are identified by their unique feature, which is their ability to absorb and retain a large quantity of water without dissolving. Hydrogels can be classified based on the method of crosslinking. Physically crosslinked hydrogels involve non-covalent interactions such as hydrogen bonding, ionic interactions, and hydrophobic interactions, offering high water content and reversible structure [131]. Chemically crosslinked hydrogels involve covalent bonds between the polymeric chains, providing improved mechanical strength and long-term stability [132]. Natural polymer-based hydrogels have recently gained attention for their biocompatibility, biodegradability, and tunable properties, leading to diverse applications including drug delivery systems, wound dressing, food packaging, water/wastewater treatment, personal care, agriculture, biosensors, etc. Research has proven that the hydrogels synthesized from natural sources exhibit satisfactory results. For instance, Ramirez Martines [133] utilized keratin and polysaccharides such as agar-agar and carboxymethylcellulose to synthesize physically crosslinked hydrogels for use as a lubricating, low-friction coating on endotracheal tubes in biomedical coating and wound healing applications. This hydrogel demonstrated superabsorbent behavior with swelling rates up to 36-fold and exhibited high biocompatibility and low friction coefficients, indicating its potential to reduce tissue damage [133]. In agriculture, biopolymeric superabsorbent hydrogels evaluated in India (i.e., Pusa Hydrogel and its kaolin derivative) based on semi-synthetic cellulose derivatives show promising outcomes. The Pusa Hydrogel applied under various irrigation regimes significantly increased soil moisture retention and soybean and wheat yields (up to 52.7% increase under rainfed conditions), demonstrating the hydrogel’s efficacy in soil amendment [134]. Natural polymer-based hydrogels have found importance in water treatment as a powerful tool for improving the engineering potential of enzymes through immobilization for contaminant degradation. Researchers at the University of Texas Austin are exploring innovative molecularly functionalized biomass hydrogels from diverse natural materials for application in water treatment. Field tests of the hydrogel systems demonstrated the ability to harvest up to 14.19 L of clean water per kilogram of sorbent daily, outperforming conventional materials. This holds great promise as a material to absorb water for effective contaminant degradation [135].

#### 2.4.4. Aerogels

Focusing on bio-based aerogels, these are primarily made from proteins and polysaccharides extracted from biomass through the sol–gel synthesis process, which is followed by solvent exchange and drying [136]. This method allows the formation of a highly porous 3D network and enables the control of some properties such as porosity, mechanical strength, and surface area. Since bio-based aerogels still have some limitations and thus cannot exhibit all the properties of their inorganic counterparts, research has currently focused on the development of hybrid aerogels that are functionally tailored to meet their applications in different fields [137]. In this modification, the addition of inorganics helps to improve their chemistry and their unique features.

However, among all the stages involved during aerogel synthesis, the drying stage has the highest influence on the properties of the aerogels. These drying methods include supercritical CO_2_ drying, which involves heating the wet gel at a temperature and pressure above the critical temperature and pressure of the liquid stored inside the gel’s pores and offers structural integrity and high porosity. Freeze drying refers to the sublimation of the solid, usually in the frozen state, which is economically cheap but has a lower surface area, and atmospheric pressure drying, which is industrially applicable but causes shrinkage or the formation of a solid film without porosity [136].

As a result of these critical features, aerogels are applicable in various fields, including tissue engineering, as a microporous scaffold [136]; in biomedicine, as drug delivery carriers (e.g., starch-based nanoporous aerogels for ketoprofen distribution) [138]; and in energy storage systems, as a material with increased surface area and improved conductivity due to nitrogen doping that enhances electrode performance [139]. Such a matrix was synthesized in the form of an N-doped porous carbon nanofiber aerogel from marine biomass waste through cellulose nanofiber pyrolysis in NH_3_ and KOH activation.

#### 2.4.5. Films/Membranes

The environmental implications of films and membranes fabricated from natural polymeric materials are substantial, offering a dual benefit of reduced reliance on fossil fuels while also addressing environmental issues related to their non-biodegradable counterparts. Natural biopolymers such as cellulose, lignin, chitosan, alginate, and starch exhibit unique mechanical, barrier, and biocompatible properties suiting a wide range of applications. Chemical modification, crosslinking, additive incorporation, and utilizing biopolymer blends can enhance film and membrane properties [140,141]. Common methods for film and membrane fabrication include solvent casting/evaporation-induced self-assembly (EISA) [141,142], doctor blade casting [142], vacuum-assisted filtration [143], phase separation methods [144], and electrospinning [145,146].

The versatilities of films and membranes are demonstrated by recent innovations presented in numerous studies. For example, the biocompatible and flexible glycine/alginate/glycerol film sensor sealed with polylactic acid and beeswax demonstrated high piezoelectric sensitivity and stability in both in vitro and in vivo settings, positioning it as a promising alternative to rigid piezoelectric materials in biomedical sensing [141]. In food packaging, Hashemi et al. [147] introduced essential oil nanoemulsions and carbon dots into gelatin–chitosan films, resulting in improved antioxidant and antimicrobial activities [147]. Advancements in membrane technologies are also notable. Hastuti et al. [148] improved the pervaporation performance of alginate membranes in terms of flux capacity, selectivity, and mechanical strength by leveraging the strong hydrophilic characteristics of cellulose nanofibers [148]. In renewable energy, de Haro et al. [149] utilized lignin-based membranes as quasi-solid-state electrolytes in aqueous dye-sensitized solar cells (DSSCs) for enhanced photostability and device lifetime by taking advantage of lignin’s UV-protective properties [149].

#### 2.4.6. Three-Dimensional Porous Scaffolds

The inherent biocompatibility, biodegradability, and, most importantly, structural similarity to the extracellular matrix (ECM) make biopolymers invaluable for the fabrication of 3D porous scaffolds. Commonly used biopolymers, e.g., collagen, gelatin, chitosan, silk fibroin, alginate, and cellulose derivatives, have demonstrated effectiveness in developing biomimetic structures that support essential cellular functions (i.e., attachment, proliferation, and differentiation) [150,151]. These scaffolds play a vital role in advancing the biomedical sector, as they can be tailored to meet the specific requirements of various therapeutic applications.

Fabrication techniques have a strong influence on the physical and biological performance of scaffolds by dictating pore structure, fiber alignment, and scaffold geometry. Common methods include lyophilization, solvent casting/particulate leaching (SCPL), phase separation, gas foaming, electrospinning, and additive manufacturing [150,152]. For instance, electrospinning produces micro- and nanofibrous scaffolds with close ECM resemblance by controlling fiber diameter and alignment, factors crucial for tissue engineering applications such as skin regeneration [153]. On the other hand, 3D printing can allow the use of thermosensitive hydrogels to fabricate scaffolds with geometries that enhance cell adhesion and differentiation [154]. Using the SCPL technique, it is possible to produce a porous chitosan–bacterial cellulose scaffold structure favoring osteoblast adhesion and differentiation [150]. Additional modifications, like sonication and crosslinking (physical or chemical), can enhance the mechanical strength and functional stability of scaffolds [155,156]. An alginate/chitosan scaffold with gold nanoparticles fabricated by Beltran-Vargas et al. [157] has the potential to be a novel implant material for cardiac tissue engineering as it favored cardiomyocyte growth and spheroid formations, while the gold additive increased cell proliferation and promoted the production of cardiac proteins [157]. Other scaffolds useful for tissue engineering include collagen/phenolic acids-based scaffolds [158], chitosan/gelatin/poly(caprolactone)/poly(lactic acid) scaffolds [154], zein/gum arabic/marigold extract/poly(ε-caprolactone) scaffolds [153], borax crosslinked chitosan/guar gum/graphene oxide scaffolds, and others [156].

### 2.5. Functional Additives in Natural Polymer-Based Biomaterials

Natural polymer-based biomaterials are often strategically incorporated with functional additives to enhance performance or improve their mechanical, chemical, and biological properties, enabling precise tailoring for multidisciplinary applications. These additives, ranging from crosslinkers to colorants (Table 1), serve as precision tools to obtain optimal performance outcomes. For instance, crosslinkers such as glutaraldehyde, epichlorohydrin, genipin, ionic salts, and others are used in hydrogel synthesis to obtain structural stability and integrity, particularly for load-bearing applications like tissue scaffolds [159]. Therapeutic bioactive molecules such as antimicrobial peptides and growth factors are incorporated to impart biomaterials with active functionalities, as applied in infection control in wound dressing [160] and natural hydrogel systems designed for the controlled release of angiogenic factors to promote vascularization [161]. In some cases, bio-derived additives may be introduced into non-bio-based materials to promote biodegradability and reactivity, aligning material properties with circular economy principles. By combining biomaterials with functional ingredients, researchers have achieved tunable degradation rates and stimuli-responsive behavior and enhanced compatibility with industrial processing methods.

## 3. Innovations in the Application of Natural-Originated Polymeric Biomaterials

**Figure 19:**
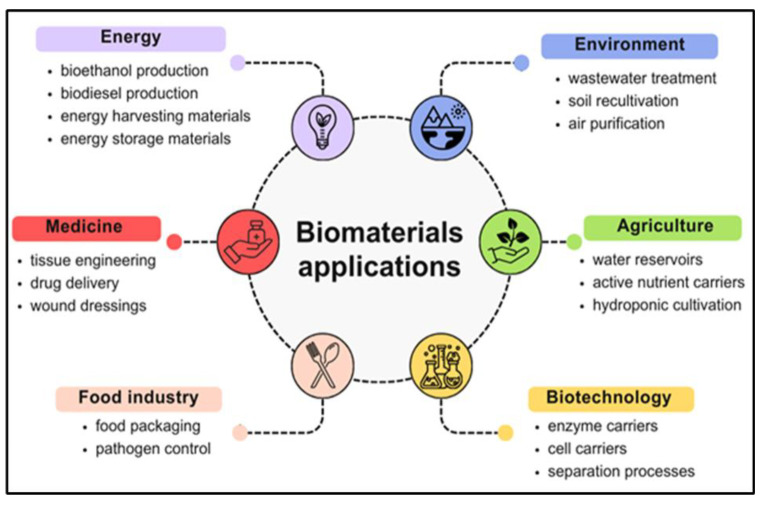
Applications of biomaterials in various sectors (created in https://www.canva.com/).

Natural-based materials have emerged as a revolutionary and sustainable type of material, driving significant advancements in medicine, the food industry, agriculture, biotechnology, and other sectors (Figure 19). The development of these biomaterials is gaining attention as a promising alternative to traditional synthetic materials, leveraging the inherent advantages of biomaterials, including adaptability, compatibility with living tissues, degradability, abundance, and cost-effectiveness.

There are several ongoing studies on the innovative use of these materials. These are emerging innovations in well-developing sectors, where the literature suggests that development rates are influenced by global trends, technological advancements, and shifting demand patterns. The environmental sector appears to be advancing quickly due to the global quest for sustainable practices, environmental regulations, and the urgent need for wastewater treatment, bioremediation, and climate change mitigation. Recent advances in biomaterials are also driving innovation in medicine, revolutionizing wound care, diagnostics, and treatment options. Table 2 offers an insight into the latest innovations in the practical development of natural-based biomaterials.

### 3.1. Energy Sector

The fabrication of lignin-derived honeycomb-like porous carbon/SiO_2_ composites for high-performance Li-ion batteries: Carbon materials play an essential role in energy systems and energy storage materials because they possess excellent properties including chemical stability, good electrical conductivity, high thermal stability, and large surface area. Among the various carbon materials, three-dimensional (3D) porous carbon materials are largely regarded as most suitable for use in the fabrication of energy storage devices like sodium-ion batteries, lithium-ion batteries, and super-capacitors due to their improved interconnected porous structure and excellent structural stability [202].

A study on the electrochemical performance of a lignin-derived honeycomb-like porous carbon/SiO_2_ composite (LHC/SiO_2_-21) with a 3D structure in a Li-ion battery highlighted the potential of biomaterials in energy storage devices [181]. The LHC/SiO_2_-21 synthesis involved a dual-template-assisted self-assembly strategy. With this method, the self-assembly of cationic quaternized alkali lignin (QAL) and anionic surfactant sodium dodecylbenzene sulfonate (SDBS) was performed in an ethanol/water solvent to form a QAL/SDBS colloidal sphere, which was subsequently combined with negatively charged SiO_2_ and taken through ultrasonic dispersion in an ethanol/water solvent, free-drying, carbonization, and alkali etching. The electrochemical performance test of the LHC/SiO_2_-21 electrode showed a specific delivered discharge and charge capacities of 2942.5 and 1070.1 mAh/g, respectively, at a constant current of a density of 0.1 A/g, with an initial coulombic efficiency (ICE) of 36.4% for the first cycle. The electrode reaches an ICE of over 98% after the 20 cycles, highlighting the electrode’s structural stability and reaction reversibility. The synthesized material was found to be suitable for use as an anode in Li-ion batteries, with a good reversible capacity of 1109 mAh/g, and a long cycle performance [181].

### 3.2. Medical Sector

The integration of collagen, fibrin, and glycoproteins into the development of organs-on-chips (OoCs) for drug testing, screening, disease modeling, and personalized medicine: Drug discovery is one of the most important aspects of the pharmaceutical industry. However, there are some shortcomings in the drug evaluation stage, including animal welfare protections, hence organ-on-a-chip technology, with the ability to simulate human organ systems, enables the drug evaluation process that includes testing for its toxicity, activity, safety, metabolism, and pharmacological efficacy; drug PK/PD modeling, disease modeling; and personalized medicine. These OoCs can be single or multiple organs on a chip and this may include lung, heart, liver, kidney, gut, bone marrow, skin, and muscles [203]. Collagen, fibrin, and glycoproteins are important in enhancing the OoC platforms. They mainly contribute to the structural and functional mimicry of the extracellular matrix (ECM), enabling cell growth, differentiation, and interaction with the microfluidic environment of OoCs. Microfluidic technology is used in the development of the OoCs where the organ-specific cells are cultured in a flask or disk with properly engineered systems that replicate the physiological conditions. Due to the transparency and biocompatibility of materials such as polydimethylsiloxane (PDMS), they are employed for chip fabrication. Biomaterials such as collagen and glycoproteins are used in 3D natural cell scaffolds to provide in vivo-like microenvironments, supporting cell attachment and morphogenesis. Fibrin simulates the extracellular matrix interactions (ECM) and glycoproteins facilitate cell–ECM communication and biochemical signaling. While OoCs have significantly advanced in the research phase, their successful conversion into commercial products is still limited. This is due to some challenges such as difficulty in the controlled application of nutrient supply and limited multiscale structures and tissue interfaces that are important for organ function. The integrated development of OoCs with artificial intelligence shows a positive change in the pharmaceutical industry including the development of OoC hardware. AI will promote convenient OoC data processing and increase the detection throughput of OoCs [204].

Oxidized sodium alginate/gelatin/halloysite hydrogel used as an injectable, adhesive, and antibacterial dressing for hemostasis: Wound care faces problems like hemorrhages and infections that may cause shock and disability and sometimes lead to death. Healing requires four main stages: (1) the hemostasis stage, being the most effective step to reduce the death rate; (2) the inflammation stage; (3) the proliferation stage; and (4) the wound structure remodeling stage. Thus, wound dressing with hemostatic and antibacterial properties is of great importance, but this is limited in conventional dressing due to insufficient mechanical strength, antibacterial efficacy, and biocompatibility. Hydrogels are polymers with 3D structures that have been applied in wound dressing due to their strong network formed because of physical or chemical crosslinked chains. Lin et al. [189] researched an oxidized sodium alginate/gelatin/halloysite hydrogel (OSA/Gel/HNTs) that has been applied for wound healing. This combines OSA and gelatin as the primary matrix combined with halloysite nanotubes. This hydrogel forms a self-crossed network through Schiff base reactions ensuring excellent mechanical properties, injectability, and skin adhesion. HNTs facilitate crosslinking, reducing the gelation time and providing antibacterial activity against some pathogens like *E. coli* and *S. aureus.* This hydrogel has been proven to also reduce clotting time, minimize bleeding, and demonstrate effective hemostatic and antibacterial properties for advanced wound dressing.

The formulation of drug-free coating functionalized with tailored collagen supports for vascular tissue healing: Coronary artery disease (CAD) is among the top cardiovascular diseases and affects a large population of human beings worldwide. A stent implantation is an effective treatment method for CAD that has shown a great evolution in its treatment, replacing the traditional surgical thoracotomy. However, some complications, such as arterial tissue damage, cause thrombus formation and acute inflammatory response. These processes together are interrelated and responsible for the excessive deposition of extracellular matrix (ECM) and the proliferation of smooth muscles, which majorly cause hyperplasia, which is a key cause for in-stent restenosis (ISR). A drug-eluting stent (DES) offers an effective way to reduce ISR by releasing drugs to inhibit smooth muscle cell proliferation. Meanwhile, delayed endothelial healing, chronic inflammation, and an increased risk of late stent thrombosis limit their efficacy and safety for use. In solving these problems, a recombinant humanized type III collagen (rhCol III) has been suggested as it offers an innovative, drug-free coating for vascular tissue healing. Through structural biology and genetic engineering, tailored biomaterial collagen is integrated as a stent coating to support vascular tissue healing. rhCol III possesses some properties like anticoagulant and cell adhesion activity, giving it the ability to retain highly adhesive fragments of Gly-Glu-Arg (GER) and Gly-Glu-Lys (GEK) of rhCol III and bypass the hydroxyproline (O) sequence, which might induce platelet adhesion and activation. A stent coating with only rhCol III coating (drug-free) possesses several effects on cardiovascular stents including low inflammatory response, reducing the LST risk, inhibiting ISR, and promoting vascular neointimal healing. Unlike DES, rhCol III naturally fosters in situ tissue repair and addresses the limitations of delayed healing and thrombosis. Hence, the biomaterial collagen prompts a promising future strategy for cardiovascular implants [190].

Sustainable collagen alternatives from engineered yeast strains for regenerative medicine and biomedical uses: Collagen is traditionally sourced from animals, which raises concerns regarding the transmission of animal diseases (e.g., bovine spongiform encephalopathies and Creutzfeldt–Jakob disease) as well as accessibility for vegan or religious consumers. ProColl is a spin-off company from Swansea University that addresses these concerns by focusing on the manufacture of humanized procollagen and collagen using engineered yeast strains for a variety of applications in the medical, cosmetic, and food industries [205]. ProColl’s main products are recombinant human procollagen Type 1 α1 and humanized Type I collagen, available as single alpha-chain or as full-chain molecules. Their procollagen has a triple helix molecular form, effectively mimicking the natural configuration of human collagen, thereby maintaining the structural and biological functionality required to retain its bioactive properties, and making it an ideal substrate for precise biomedical applications like regenerative medicine, cell therapy, and coatings for medical devices [206].

Leveraging antimicrobial compounds from berry extracts incorporated into nanocellullose films for surgical and wound care solutions: Berries are known for containing antimicrobial compounds, such as ellagitannins, that have been found to likewise be effective on human skin. These antimicrobial phenolic compounds can be sustainably sourced by utilizing the by-product of berry juicing, namely the berry press cakes, which contain the berry skin and seeds. Among the various wild berries tested, raspberry has been identified as the most suitable raw material for large-scale production due to its abundant supply. VTT Technical Research Centre of Finland’s patented InnoBerry Technologies™ manufacturing method involves dry and wet fractionation technologies followed by a sustainable hydrothermal extraction method that avoids the use of harmful solvents. The berry extract product can potentially replace artificial preservatives and nano-silver compounds in cosmetic and wound-care products [184]. More notably, it has been demonstrated to be effective in eliminating pathogens such as methicillin-resistant *Staphylococcus aureus* (MRSA), a bacterium with a global prevalence of 14.69% [207]. This is particularly interesting due to MRSA’s antibiotic resistance characteristic, which complicates treatment and can potentially be life-threatening, especially when acquired during surgeries. The antimicrobial extract developed by VTT is available in several forms and claims that it can be used as a surgical spray, cream, transdermal patch, or wound dressing. A nanocellulose film, a highly biocompatible material, can be utilized for skin applications by saturating its surface and pores with berry extract, thereby ensuring that the antimicrobial compounds are readily available rather than being trapped within the fiber network, allowing for maximum efficacy.

### 3.3. Environmental Sector

Enhancing Solar Still Efficiency with Snail Shell Biomaterials for Sustainable Water Purification: This innovation, as applied in the environmental sector, explored the integration of snail shell biomaterials into solar still technology to improve freshwater production. In the design of the solar still system, snail shells were utilized as absorbers to enhance the evaporation process by capturing solar energy effectively to increase the temperature in the basin. The performance of the system was quantified as snail shell solar still system (SSSS) productivity, demonstrating a 6.8% increase in hourly productivity compared to conventional solar still systems (CSSS) as well as cumulative productivity of approximately 2.28 kg/m^2^. The study also evaluated the energy efficiency of the SSSS, reporting a 24.1% efficiency, which was slightly higher than that of the CSSS (23%). This underscores the potential of snail shell biomaterial for sustainable water purification by harnessing solar energy. Considering its abundance and low cost, it is an environmentally friendly alternative to synthetic materials. Nonetheless, potential challenges include preparation, durability, and maintenance, hence the need for design optimization to maximize the effectiveness of the snail shells in the solar still. Overall, the research underscores the viability of this innovative approach to enhance solar desalination processes and contribute to sustainable water management solutions [192].

Eco-Friendly Cellulose Aerogel Composites for Efficient Dye and Oil Removal from Wastewater: This innovation, as applied in the environmental sector, also explores the synthesis and usage of cellulose aerogel composites derived from pineapple leaf fibers and cotton waste to address the pressing issue of wastewater pollution caused by dyes and oils. The technology utilizes the sol–gel method in an alkali–urea solution, where cellulose fibers are dissolved in the alkali–urea solution (typically NaOH-Urea) to break down the structure, allowing the formation of a homogenous solution and subsequent formation of a gel under suitable conditions, followed by freeze-drying to create aerogels with remarkable properties, including low density (0.053–0.069 g/cm^3^) and high porosity (nearly 95%). The mechanical performance exhibited had between a 5 and 9 times higher Young’s modulus compared to previous aerogel composites, underscoring the feasibility of using agricultural waste to produce effective water treatment materials and the potential of these cellulose aerogel composites to contribute to sustainable environmental solutions [193].

Innovative strategies in PHA production by utilizing greenhouse gases for sustainable bioplastics: Polyhydroxyalkanoates (PHA) are biopolymers that serve as sustainable alternatives to fossil fuel-based plastics, as these have comparable plastic properties like high elasticity, tensile strength, and air resistance, with the added advantages of biocompatibility and biodegradability. PHAs serve as microorganisms’ energy and carbon storage and are produced via fermentation of sugars and fatty acids from waste feedstocks. Despite their advantages, PHA production is greatly hindered by the high production cost, with carbon sources accounting for 35–40% of the total cost [191]. Utilizing greenhouse gases (GHGs) like methane (CH_4_) and carbon dioxide (CO_2_) as carbon sources offers several benefits of carbon sequestration, promotes a bio-based circular economy, and reduces costs. This is an underexplored field, with fewer than 300 publications indexed in the Web of Science Core Collection (keywords used: Polyhydroxyalkanoates/PHAs/PHB, carbon dioxide/CO_2_, methane/CH_4_, fermentation/bioconversion for analyzing PHAs production from CO_2_, and fermentation/bioconversion for analyzing PHA production from CH_4_); however, it is gaining traction, with over 80% of these studies published within the last decade [199]. Challenges identified include low productivity, limited microbial species, and inefficient gas-to-liquid transfer rates, and may be addressed by stress-induced PHA production, the use of thermophilic microbial communities, and advancements in co-culturing and genetic engineering. Techniques such as using water-in-oil emulsions have improved PHA synthesis, significantly increasing microbial growth and PHA accumulation. Mixed carbon sources, like biogas, also enhance polymer properties, particularly poly(3-hydroxybutyrate) (P(3HB)), a widely studied PHA. This technology is exemplified by the company Mango Materials, which partners with and capitalizes on methane-producing facilities such as landfills and wastewater treatment plants. The company uses a consortium of methanotrophs to primarily produce P(3HB) pellets that have been optimized for versatility, finding applications in injection molding, fiber extrusion, additive manufacturing, and other plastic-substitute uses. Within approximately six weeks, the material disintegrates due to microbial activity in the ocean, without leaving harmful residues. This makes the material environmentally sustainable and suitable for reducing plastic pollution in aquatic ecosystems [208].

### 3.4. Construction Sector

Rigid flame-retardant foam as a building material synthesized from bio-based sucrose-furanic resin: Bio-based foams present a promising alternative to conventional foams widely used as insulation materials in different sectors, particularly the construction and building industries. Bio-based foams offer some advantages over fossil-based foams including sustainability and low cost [15]. The flame-retardant properties of sucrose-furanic resin material have been studied by Dong et al. [196]. The study synthesized sucrose-based foams by incorporating crosslinking agents: glyoxal and furfuryl alcohol into sucrose to form a sucrose-furan-glyoxal (SFG) resin, and the subsequent addition of ammonium dihydrogen phosphate (ADP) and azodicarbonamide (AC) to obtain SFGA foam. Analytical results showed that the SFGA foam has a temperature of heat resistance index of 116.40 °C and a limiting oxygen index (LOI) of 43.3% which meets the building B1 standard (LOI ≥ 32%). The SFGA foam exhibited excellent thermal insulation capabilities, making it suitable for use as a flame retardant. Compared to conventional flame retardants, which emit harmful gases such as carbon monoxide and hydrogen cyanide during combustion, the SFGA foam emits acetic acid, carbon dioxide, and oxanes. A biodegradability test also shows that the foam is biodegradable, attaining a weight loss of 2.7% after 30 days of being buried in the soil. Overall, the SFGA foam attained the UL-94 V-0 flame-retardant classification and hence can be used as an alternative flame retardant [196].

The synthesis of chitin/chitosan biocomposite foams for sound absorption using chitins from different organisms: Wachsmann et al. [197] conducted a comprehensive study on the sound absorption properties of chitin/chitosan biocomposite foam. The study implored a “shake and bake” method of synthesizing chitin/chitosan-based foam by mixing different amounts of chitin of the fungus *A. niger* α-ChiGL (α-chitin with glucan), snow crab *C. opilio* α-ChiSC (α-chitin), or squid *C. coleoidea* β-ChiSQ (β-chitin) with a solution of chitosan and starch to form a slurry, which was subsequently mixed with ammonium carbonate, generating a carbon dioxide as the foaming agent in an acidic medium. A mechanical test analysis showed that the chitin foams exhibit mechanical properties comparable to medium-density (MD) flexible polymer foams, with α-chitin similar to low-density and β-chitin similar to medium-density polymers. The study also reported that the synthesized foams recorded a total heat release (THR) of 16.96−17.96 kJ/g and 16.52–17.14 kJ/g for the α-chitin-based foam and β-chitin-based foams, which are comparatively lower than a commercial insulation material, with expanded polystyrene (EPS) and extruded polystyrene (XPS) having a THR of 24−45 kJ/g. These findings highlight the superiority of the chitin-based foams in terms of fire safety. The chitin-based foams exhibit good sound absorption coefficient maxima in a range of 0.79–0.94, corresponding to 600−2000 Hz, making them suitable for use in daily street noise (sound cancellation in the range of 700−1300 Hz). The sound absorption properties of the chitin foams are comparable to existing sound-absorber materials such as Rockwool (0.9, 500 Hz) and other absorber panels (0.90, 1000 Hz) [197,209].

### 3.5. Textile Sector

Promoting Green Fashion through State-of-the-Art Sustainable Leather Alternatives: The textile industry is one of the most environmentally harmful industries, accounting for 20% of water pollution and 10% of Greenhouse Gas (GHG) production globally. Additionally, it reportedly utilizes an average of 400 m^2^ of land, 9 m^3^ of water, and 391 kg of raw materials while generating 270 kg of CO_2_ emissions per year per capita. Its contribution to environmental pollution is not limited to the manufacturing process, but also throughout its lifecycle. During use, microfibers are released during washing, and less than 1% are recycled, with the remainder often ending up in landfills, thereby exacerbating waste and pollution concerns. Despite this, the demand for textiles continues to rise, with projections indicating a global production of 145 million tonnes by 2030, driving the demand for alternative, animal and petrochemical-free biotextiles [210].

Companies such as TômTex, Modern Synthesis, and MYCOWorks^TM^ are driving innovation in sustainable biotextiles. TômTex creates 100% certified bio-based textiles that have a much lower environmental impact by requiring only 0.1% of the water used in conventional leather production and 80–90% less GHG emissions. They offer biodegradable, petrochemical-free, and customizable products made of sustainably sourced chitosan from mushrooms and shrimp and crab shells for their TômTex Series M^TM^ and Series WS^TM^, respectively [198]. ModernSynthesis creates bacterial nanocellulose-based textiles from agricultural residues, offering lightweight, leather-like, biodegradable materials with high tensile strength (about 8 times that of steel) through a closed-loop system. Natural fibers are utilized as microbial scaffolds, providing a framework for microbes to grow and produce nanocellulose during fermentation. As this process occurs, the textile material forms around the scaffold, creating a structured and functional product [199]. MycoWorks™ creates leather-like materials with specific strength, performance, and aesthetic requirements by leveraging its patented Fine Mycelium™ technology, which involves the precise engineering and monitoring of mycelium growth. The biomaterial still undergoes tanning and finishing processes, but the company employs a proprietary chrome-free tanning and dyeing technology designed to minimize environmental impact [200].

Harnessing genetic engineering for creating pigmented biotextiles from bacterial cellulose: Genetic engineering can be utilized to further enhance the sustainability of biotextiles made from bacterial cellulose by creating microbial strains capable of producing self-pigmenting materials. An example of this is the genetic modification of *Komagataeibacter* rhaeticus performed by Walker et al. [201] to express the *Tyr1* tyrosinase gene obtained from *Bacillus megaterium*. This enzyme catalyzes the oxidation of L-tyrosine into dopaquinone, which eventually converts into eumelanin, the pigment responsible for dark colors like brown and black. Two Tyr1 expression strains were produced, namely, the plasmid-based *K. rhaeticus ptyr1* and chromosomally integrated *K. rhaeticus ctyr1*. A two-step process was developed involving the culturing of the Tyr1-expressing bacteria under normal acidic growth conditions followed by melanin synthesis, which requires a specific melanin development buffer with basic pH. The melanated pellicle production was successful, with the darkest color observed after 19 h. It was found that adjusting the time and L-tyrosine concentration in both the culture medium and melanin development buffer allowed for the creation of various shades of brown and black. The melanin pigment proved resistant to sterilization by high-pressure steam and ethanol but not to oxidizing agents like sodium hypochlorite. It withstood the water spotting test and showed high stability with no visible discoloration. Moreover, the pigment persisted, and minimal changes were observed even after 42 months of testing. Notably, it can likewise be patterned using light sources. Testing revealed no significant differences in tensile strength or porosity between melanated and unmelanated pellicles. However, melanated pellicles showed increased hydrophilicity, with the contact angle dropping from 47° to 28°. This means that waterproofing may be required, either by using natural materials or through further genetic modifications to alter the structure or create a hydrophobic layer. Future research could explore the creation of other pigment variations [201].

## 4. Future Development Trends

The advancement of biomaterials based on natural polymers seeks to maximize the industrial revolution in various fields such as biomedicine, sustainable materials, environmental biotechnology, and advanced manufacturing. As the demand for biodegradable, biocompatible, and functionally adaptive materials is rapidly increasing, it has become imperative for research to focus on enhanced fabrication methods, hybrid material innovation, and diversifying applications. According to the current literature prognosis [211], the rapid demand for bio-based products in the global market is driven by regulatory policies, environmental concerns, and technological advancements. Production capacity in 2023 increased to 4.4 million tonnes, representing 1% of the total polymer market, with a compound annual growth rate (CAGR) of 17% projected until 2028. Asia and North America are specifically experiencing the fastest growth, where companies are investing in sustainable polymer production [211].

**Figure 20:**
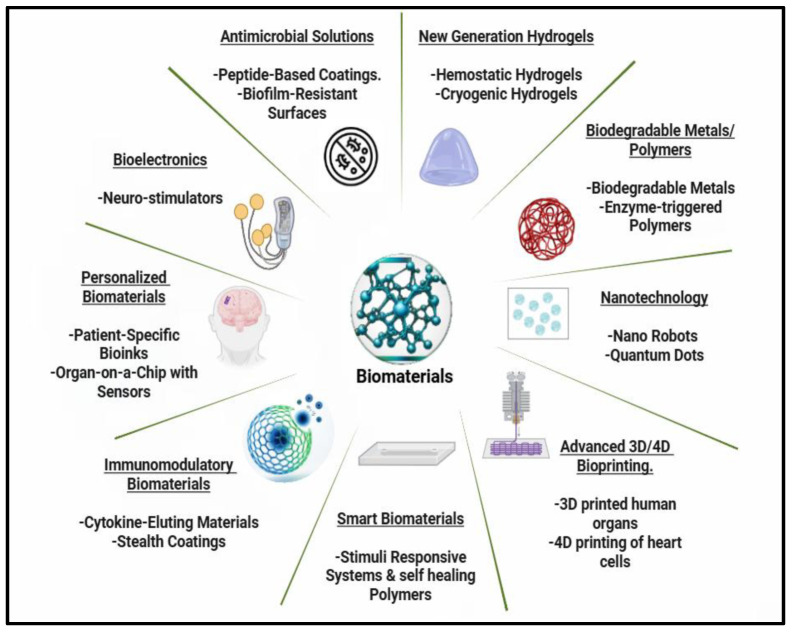
Observed biomaterial trends [218,219,220,221,222,223,224,225] (Created in BioRender. Amponsah, O. (2025) https://BioRender.com).

Significant trends observed recently include the integration of advanced fabrication technologies such as 3D/4D bioprinting, electrospinning, bioelectronics, smart biomaterials, personalized biomaterials, new generation hydrogels, antimicrobial solutions, immunomodulatory biomaterials, biodegradable polymers/metals, and nanotechnology, which enable the precise structuring of biomaterials for various applications [212,213,214,215,216,217,218,219,220,221,222]. Natural-based hydrogels like chitosan, sodium alginate, hyaluronic acid, and cellulose have gained prospects due to their high water content, biocompatibility, and similarity to the extracellular matrix (ECM), making these biopolymers ideal for tissue scaffolds, biosensors, and controlled drug release systems [222,223]. It was found by Barhoum et al. [224] that bio-based hydrogels are being developed with stimuli-bioresponsive properties, enabling the production of new-generation materials applied as implantable, wearable, and disposable biosensors for medical diagnostics [224]. Figure 20 illustrates various significant shifts in the recent utilization of biomaterials, demonstrating their growing dominance in contemporary society.

Key development trends include the development of transformative nanorobots through fabrication techniques such as two-photon polymerization (TPP) to enable precise 3D micro/nanostructures with submicron resolution, critical for creating biodegradable, stimuli-responsive robots for targeted drug delivery [218] and the development of new elastic materials for soft living tissue 3D printing. Researchers of Northeastern University Bioengineering recently patented a new elastic hydrogel material for such applications and made a significant assertion that a breakthrough is expected to lead to the 3D printing of blood vessels and human organs very soon [225]. Delgado-Puyol et al. [219] also discuss a shift toward stimuli-responsive biomaterials, particularly hydrogels and nanogels, laying emphasis on personalized medicine through 3D-printed and injectable systems, patient-specific bioinks, and organs-on-chips with sensors that adapt to patient-specific anatomical or pathological conditions. These biomaterials enable precise drug release via pH, temperature, or enzymatic triggers [219]. Biodegradable metallic materials have also seen a significant rise in medical implants by combining mechanical strength akin to human bone with in vivo degradability, eliminating secondary removal surgeries. These metallic materials including magnesium, zinc, and iron alloys are being investigated thoroughly with innovations focusing on surface modifications (e.g., fluoride coatings for Mg alloys) to control corrosion and leveraging 3D printing to fabricate patient-specific implants, despite challenges in surface roughness and structural precision [217].

Another promising development in bioelectronics is the development of neurostimulators. Implantable neurostimulators like spinal cord stimulators are being developed for chronic pain management by delivering electrical pulses to specific nerves to disrupt pain signals and provide relief to patients. Beyond that, they hold enormous promise in treating neurological disorders. For instance, brain–machine interfaces and neuromodulation devices are designed to address conditions like epilepsy, depression, and neurodegenerative diseases through direct interaction with the nervous system to either stimulate or inhibit neural activities to restore proper function. The future of healthcare is becoming more reliant on unlocking bioelectronic device advancements [212]. Despite these advancements, several challenges remain, as highlighted earlier. Biomaterials based on natural polymers are often limited by poor mechanical strength, batch-to-batch variability, and rapid degradation, which make it challenging for applications including load-bearing medical devices and long-term implants [223]. Strategies such as chemical crosslinking, nanocomposite reinforcement, and enzymatic modifications are expected to take over since they enhance stability and durability [222]. However, chemical crosslinking, which utilizes chemical agents such as acids and glutaraldehyde, may have a negative impact on the environment when released in quantities. Additionally, cost-effective mass-production techniques are needed to bridge the gap between laboratory research and industrial-scale manufacturing. Nevertheless, with consistent improvements in processing techniques, material engineering, and policy support from relevant stakeholders, these biomaterials will be central to next-generation materials for various eco-friendly industrial applications and functional biotechnological approaches.

## Figures and Tables

**Table 1:** Some common functional additives for enhancing biomaterial performance.

Additive Type	Examples	Specific Role	Outcome	Reference
Crosslinkers	Glutaraldehyde Genipin Epichlorohydrin 1,4-Butanediol diglycidyl ether (BDDE) N,N′-methylenebisacrylamide Ethylene glycol dimethacrylate Poly (ethylene glycol) diacrylates	Formation of chemical or covalent bonds between polymer chains to enhance stability and mechanical strength.	Enhance structural integrity, tunable degradation rates, reduce rapid turnover. Toxicity concerns with synthetic crosslinkers.	[162,163]
Bioactive molecules	Polynucleotides (PN) Mannitol Growth factors Antimicrobial peptides Retinol Ascorbic acid Ferulic acid	Stimulate biological responses such as antioxidation, infection control, drug release, etc.	Enhanced tissue regeneration, antioxidative protection, infection control, vascularization, and prolonged biomaterial life.	[164]
Reinforcing agents	Nanocellulose Nanoclay Nanoparticles	Increase mechanical strength, stiffness, surface area, biocompatibility, water retention.	Improved load-bearing capacity, stress resistance, hydrophilicity, and durability.	[165,166]
Plasticizers	Glycerol, ethylene glycol (EG) Diethylene glycol (DEG) Triethylene glycol (TEG) Triethanolamine (TEA) Sorbitol	Reduce brittleness and increase flexibility.	Enhanced elasticity, processability, mechanical and barrier properties.	[167,168]
Fillers	Hydroxyapatite (HA) Tricalcium phosphate (TPA) Bioactive glass Silica nanoparticles	Modify texture, mechanical properties, and bioactivity.	Increased hardness, density, compressibility, osteoconductivity, and bioactivity. HA improves bone metabolism and has antibacterial effects.	[169,170]
Conductive agents	Polypyrrole Polyaniline Polythiophene Graphene oxide Carbon black	Impart electrical conductivity into the material.	Enable stimulus-responsive behavior and biosensing applications.	[171]
Flame retardants	Phosphates (e.g., Diethyl(hydroxymethyl)phosphonate and triphenyl phosphate (TPHP)) Halogenated compounds (Polybrominated diphenyl ethers (PBDEs))	Improve thermal stability and reduce flammability.	Enhance processing and application safety for wearable sensors and textiles.	[172,173]
Antistatic agents	Quaternary ammonium compounds (benzylalkyldimethyl ammonium compounds (BACs), Alkyltrimethyl ammonium compounds (ATMACs) Dialkyldimethyl ammonium compounds (DADMACs))	Reduce static charge accumulation in biomaterials.	Enhance handling and comfort in wearable applications.	[174]
UV stabilizers	Hindered amine light stabilizers (HALS) Benzophenone derivatives (Benzophenone-3 (BP-3), Benzophenone-12 (BP-12))	Benzophenones absorb harmful UV light and dissipate as harmless thermal energy. HALS functions by scavenging free radicals.	Prolonged material lifespan and stability under sunlight exposure.	[175,176]
Colorants	Natural dyes (anthocyanins, *Indigofera tinctoria*, *Rubia tinctorum*, *Curcuma longa*, *Lawsonia inermis*, *Haematoxylum campechianum*, etc.) Synthetic dyes (Tartrazine (E102), Indigo Carmine (E132), Allura Red (E129), Sunset Yellow (E110), etc.)	Provides aesthetic appeal and visualization.	Improved product appearance and visualization.	[177]

**Table 2:** Table showing some innovative applications of biomaterials.

Sector	Biomaterial Type	Innovation	Reference
Energy	Cellulose	A plant-like battery, also called a biodegradable battery.	[178]
	Gelatin hydrogel	Biodegradable primary zinc–molybdenum (Zn−Mo) battery with gelatin hydrogel electrolyte.	[179]
	Algal biopolymers	Usage as electrodes, binders, electrolytes, and separators in batteries (green battery cycle—Li_x_C_6_ and LiFePO_4_).	[180]
	Lignin-derived carbon	Mesoporous lignin-derived honeycomb-like porous carbon/SiO_2_ composites for high-performance Li-ion battery.	[181]
Medicine	Wild berry extract and nanocellulose	Antimicrobial skin sprays and surgical dressings.	[182]
	Poly L-lactide-co-glycolide copolymer (PLGA)	Production of polylactic acid (PLA) and polyglycolic acid (PGA) for the synthesis of bioabsorbable implants applied in orthopedics, cardiovascular interventions, and tissue engineering to provide temporary support while facilitating tissue regeneration.	[183]
	Collagen, fibrin, and glycoproteins	Integrated into the development of organs-on-chips (OoCs), which have great implications for drug testing, screening, disease modeling, and personalized medicine.	[184]
	Lipids-based membrane shells	Integrated micro-/nano-drug delivery system based on magnetically responsive phase-change droplets for ultrasound theranostics.	[185]
	Cellulose nanocrystals from rice husks	Reinforced polymethyl methacrylate (PMMA) nanocomposites for dental application.	[186]
	Nanocellulose	Nanocellulose membrane that propels artificial lung devices.	[187]
	Hyaluronan	Advanced hydrogel designed for precise ocular delivery.	[188]
	Alginate and gelatin	Oxidized sodium alginate/gelatin/halloysite hydrogel used as an injectable, adhesive, and antibacterial dressing for hemostasis.	[189]
	Collagen	Formulation of drug-free coating functionalized with tailored collagen supports for vascular tissue healing.	[190]
Environment	Polyhydroxyalkanoate (PHA) from methanogens	Concurrent carbon capture (CO_2_ and CH_4_) and utilization by methanogens for PHA production.	[191]
	Snail shells	Snail shell biomaterials in solar still for clean water production.	[192]
	Cellulose	Cellulose from pineapple leaf fibers and cotton waste are used in making aerogel composites for the removal of dyes and oil in wastewater.	[193]
	Microalgae biomass	Integrated carbonate-based carbon capture and algae biofixation systems.	[194]
Construction	Mycelium based composite (MBC)	MBC as load-bearing masonry components in construction.	[195]
	Sucrose	Rigid flame-retardant foam as a building material synthesized from bio-based sucrose–furanic resin.	[196]
	Chitin and chitosan	Chitin/chitosan composite foam for sound absorption.	[197]
Textile	Chitosan from mushroom and shrimp shells; bacterial cellulose; mycelium	Animal and petrochemical-free biotextiles as an alternative to conventional leather and textiles.	[198,199,200]
	Melanated bacterial cellulose from genetically engineered *Komagataeibacter rhaeticus*	Used as a self-pigmented biotextile.	[201]
